# TSLP Promotes Induction of Th2 Differentiation but Is Not Necessary during Established Allergen-Induced Pulmonary Disease

**DOI:** 10.1371/journal.pone.0056433

**Published:** 2013-02-20

**Authors:** Sihyug Jang, Susan Morris, Nicholas W. Lukacs

**Affiliations:** Department of Pathology, The University of Michigan, Ann Arbor, Michigan, United States of America; French National Centre for Scientific Research, France

## Abstract

Thymic stromal lymphopoietin (TSLP) has been implicated in the development of allergic inflammation by promoting Th2-type responses and has become a potential therapeutic target. Using *in vitro* T cell differentiation cultures we were able to validate that TSLP played a more critical role in the early development of Th2 immune responses with less significant enhancement of already developed Th2 responses. Adoptive transfer of naive DO11.10 ovalbumin-specific T cells followed by airway exposure to ovalbumin showed an early impairment of Th2 immune response in TSLP−/− mice compared to wild type mice during the development of a Th2 response. In contrast, transfer of already differentiated Th2 cells into TSLP−/− mice did not change lung pathology or Th2 cytokine production upon ovalbumin challenge compared to transfer into wild type mice. An allergen-induced Th2 airway model demonstrated that there was only a difference in gob5 expression (a mucus-associated gene) between wild type and TSLP−/− mice. Furthermore, when allergic animals with established disease were treated with a neutralizing anti-TSLP antibody there was no change in airway hyperreponsiveness (AHR) or Th2 cytokine production compared to the control antibody treated animals, whereas a change in *gob5* gene expression was also observed similar to the TSLP−/− mouse studies. In contrast, when animals were treated with anti-TSLP during the initial stages of allergen sensitization there was a significant change in Th2 cytokines during the final allergen challenge. Collectively, these studies suggest that in mice TSLP has an important role during the early development of Th2 immune responses, whereas its role at later stages of allergic disease may not be as critical for maintaining the Th2-driven allergic disease.

## Introduction

Asthma is a chronic inflammatory disease characterized by reversible airflow obstruction, over-production of mucus, and airway hyperresponsiveness [1], [2]. Previous reports demonstrated that T-helper type 2 (Th2) cells play a critical role in the pathogenesis of asthma by releasing type 2 cytokines, such as IL-4, IL-5, and IL-13 [3]–[4]. Recently many studies have demonstrated that thymic stromal lymphopoietin (TSLP) is involved in initiating Th2 differentiation as well as in allergic inflammatory responses [5]–[7]. TSLP is expressed by epithelial cells at barrier surfaces, activated bronchial smooth muscle cells, and activated mast cells [8]. The TSLP receptor consists of IL-7 receptor alpha chain and a unique TSLP receptor chain [9]. TSLP signaling appears to be dispensable for immune system development, as mice lacking TSLPR are normal [10]. TSLP receptor −/− (TSLPR −/−) mice fail to develop an allergic response in the ovalbumin induced allergic murine model, whereas mice specifically expressing TSLP transgene in the lung had an enhanced allergic response to innocuous antigens [5], [6]. These data support an association between TSLP and the development of allergic disease. In humans, TSLP expression is elevated in the lesion skin of atopic dermatitis patients and in the lungs of asthmatic patients [11]–[12]. TSLP has been shown to promote the ability of human DCs to polarize naïve T cells into Th2 cells by up-regulating OX40L on DCs in the absence of IL-12 [11] [13]. In addition to its role on DCs, it was demonstrated that TSLP directly alters murine T cells and promotes Th2 differentiation via induction of IL-4 transcription [14]. TSLP has further been shown to be important for development of basophil populations that may also contribute to allergic and inflammatory diseases [8], [15], [16]. The role of mast cells during TSLP-mediated responses further extenuates the TSLP associated responses during allergy [17], [18], [19]. Thus, TSLP has been clearly demonstrated as a key factor for the development of allergic disease. However, several studies have also linked TSLP with regulatory T (Treg) cell development, therefore potentially functioning in at least some instances as a disease modifying molecule [20], [21], [22].

In this study, we examined the role of TSLP on dendritic cells and on T cells during the early phase of Th2 differentiation in vitro and in adoptive T cell transfer experiments. To further investigate its role on allergic reaction, we used a clinically relevant cockroach antigen-induced allergic model. Furthermore, we examined the efficacy of TSLP blockade during late stage allergic responses. Our data demonstrate that TSLP enhanced development of Th2 immune responses, but had a little effect on established allergic disease.

## Materials and Methods

### Mice

BALB/c and DO11.10 mice were purchased from The Jackson Laboratory (Bar Harbor, ME). TSLP−/− mice [23] and anti-TSLP monoclonal antibodies (M702) [16] were graciously supplied by Dr. Michael R. Comeau at Amgen (Thousand Oaks, CA). All animal work was reviewed and approved by the University of Michigan animal welfare review committee and was conducted according to relevant national and international guidelines.

### Airway Response

Airway hyperreactivity was assessed as previously described using direct ventilation methodology and airway resistance measurements calculated from direct airway resistant changes [24]. Briefly, mice were anesthetized, intubated via cannulation of the trachea, and ventilated with a ventilator (0.3 ml tidal volume, 120 breaths/min). Airway hyperreponsiveness (AHR) was measured using mouse plethysmography and software for calculation of the measurements (Buxco, Wilmington, NC, not PENH). After baseline measurements, mice were injected i.v. with 7.5 ug methacholine (Sigma-Aldrich, St. Louis, MO), and the peak airway resistance was recorded as a measure of AHR.

### Cockroach Ag Model

Cockroach allergen extract (CRA, Hollister-Stier, Spokane, WA) sensitization was performed as previously described [25], [26], [27]. Briefly, female mice were sensitized with a 1∶1 mixture of CRA and incomplete Freund’s adjuvant (IFA) (Sigma-Aldrich), both s.c. and i.p. on day 0. In the chronic CRA model, at days 14, 18, 22, and 26, mice were locally challenged with CRA by i.n. route followed by two more i.t. challenges on days 30 and 32. In vivo i.p. administration used 300 ug of control rat immunoglobulin G (Jackson Immunoresearch Laboratories, West Grove, PA) or anti-TSLP ab on day 30 and 32 just before i.t. delivery of CRA or on day 0 and 3 depending on the procedure. In a short-term CRA model, two protocols were used. First in an i.p./s.c. sensitized short-term model, the sensitization was the same as in the chronic CRA model, but mice were challenged with CRA by i.n. route on day 8 and day 10. In the second short-term model, mice were injected with CRA by i.n. every other day (six times) until day 10. Development of a sensitization CRA model, mice were injected with CRA intranasally every other day three times. One day after final i.n. challenge, mice were euthanized to collect lungs and lymph nodes.

### Preparation of DCs and CD4 T Cells

For generation of bone marrow-derived dendritic cells (BMDCs), bone marrow cells were seeded in T-150 tissue culture flasks in RPMI 1640 based complete medium with GM-CSF (15 ng/ml)(R&D Systems). On day 3, GM-CSF was supplemented into cultured cells (15 ng/ml). On day 6, loosely adherent cells were collected and purified with anti-CD11c beads using a magnetic column (Miltenyi Biotec, Auburn, CA). CD4 splenic T cells were purified by negative selection using CD4 T cell Isolation Kit (Miltenyi Biotec). For isolation of lung CD11c cells, lungs were digested with collagenase, CD11c cells were isolated by using anti-CD11c beads, and sorted to increase the purity of CD11c positive cells. Alveolar macrophages were excluded by using their auto-fluorescence properties.

### In vitro T Cell Activation

To see the effect of TSLP on DCs, BMDCs or lung CD11c cells were incubated with ova peptides (10 ug/ml) (Peptides International Inc., Louisville, KC) and either presence or absence of TSLP (15 ng/ml) overnight. On the next day these DCs were washed extensively and co-cultured with DO11.10 CD4 T cells for 48 hours for BMDC co-cultures and 72 hours for lung CD11c co-cultures. To examine the effect of TSLP on naïve T cells, DO11.10 CD4 T cells were co-cultured with BMDCs with ova peptides and +/− TSLP for 48 hours. To determine the effect of TSLP on Th2 cells, DO11.10 CD4 T cells and irradiated BMDCs were incubated with ova peptides in Th2 biased conditions (10 ng/ml of IL-4, 10 ug/ml of anti-IFNgamma and 10 U/ml of IL-2). On day 3 and 5, these cultures were split into 1∶3 ratios in 40 U/ml of IL-2 containing medium. On day 8, cells were separated on a Ficoll-paque gradient to remove dead cells, then stimulated with BMDCs and ova peptides±TSLP for 48 hours. The supernatants from these cultures were assayed for cytokines by Bioplex (Bio-Rad Laboratories, Hercules, CA).

### Histology and RT-PCR

Right lobes from mice were removed, fixed in formalin, and stained with periodic acid-Schiff (PAS). Total RNA was isolated from lower-left lobes of lungs using TRIzol (Invitrogen, Carlsbad, CA). Real-time PCR was performed on cDNA using primers. Primers and probes were purchased from Applied Biosystems (Carlsbad, CA). GAPDH was used as an internal control and lungs of naïve mice were assigned an arbitrary value of 1.

### Broncoalveolar Lavage Fluid

Bronchoalveolar lavage fluid was collected by inserting an 18-gauge needle into the traceha of euthanized mice and flushing with 1 ml PBS. Resulting cells were spun, counted and stained with Diff-Quick reagents (Siemens, New York, NY).

### Ovalbumin Injection and Adoptive Transfer Experiment

**Figure 1:**
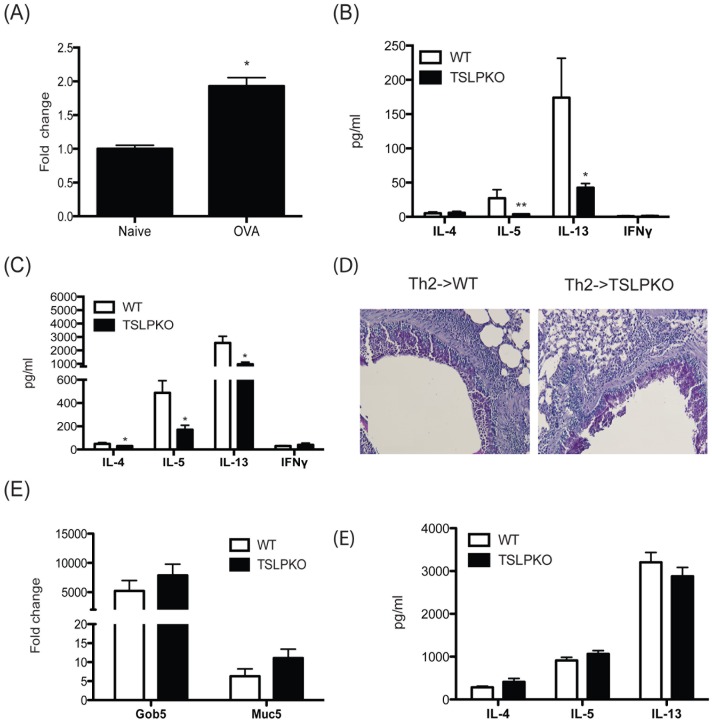
TSLP enhances development of Th2 immune responses during initiation of early phase of Th2 responses, but is dispensable on established Th2 responses. Wild type and TSLP−/− mice were exposed with ovalbumin (10 µg) by intranasal delivery every other day until day 5. *(A)* TSLP mRNA expression in the lungs of mice sensitized for 6 days with ovalbumin. (B) On day 6, supernatants from allergen-restimulated lung draining lymph nodes were analyzed for cytokines by Bioplex. (C) Naïve DO11.10 CD4 T cells were adoptively transferred into wild type or TSLP−/− mice and these mice were challenged with ovalbumin (5 µg) every other day until day 5. On day 6, lung draining lymph node cells were allergen-restimulated and supernatants analyzed by Bioplex. In a third set of animals Th2 skewed DO11.10 CD4 T cells were adoptively transferred into wild type and TSLP−/− mice and exposed to ovalbumin for 5 days as above. Subsequently, lungs from mice that received Th2 cells and ovalbumin were (D) stained with PAS and (E) assayed for gob5 and muc5ac expression by qPCR. Fold changes were calculated upon comparison to control lungs from naïve animals. (F) Cytokines were analyzed by Bioplex from supernatants of restimulated lung draining lymph node cells from mice with Th2 cell transfer. Data represent mean±SEM from 4 to 5 mice/group. Lymph nodes from naïve mice challenge with allergen elicit no detectable response.

Naïve DO11.10 T cells (4×10^6^ cells) or Th2 cells (4×10^6^ cells) were transferred via tail vein injection into mice on day 0. On day 1, 3, and 5 5 ug of ovalbumin (Grade V, Sigma-Aldrich) was injected by i.n. route. On day 6 mice were euthanized and lungs and lymph nodes were harvested. The same protocol for ovalbumin injections was used for Figure 1A except 10 µg of ovalbumin was used.

### Restimulation of Lung Draining Lymph Nodes

Single cell suspensions of lung draining lymph nodes were plated at a concentration of 5×10^6^ cells/ml onto a 96-well plate and restimulated with either CRA (3 ug/ml) or ovalbumin (150 ug/ml) for 48 hours, and supernatants were harvested for cytokine determination. Cytokines were quantified using a Bioplex cytokine assay.

### Statistics

Statistical significance was determined by one-way ANOVA with Newman-Keuls post-test. Significant differences were regarded as * = *p*<0.05, ** = p<0.01.

## Results

### TSLP has a Critical Role during the Initiation of Th2 Responses, but not on Established Th2 Responses

Previous reports demonstrated that TSLP plays a critical role in Th2 type allergic inflammation, but the precise roles on the allergic disease remain to be elucidated. To examine whether the presence of TSLP at the early phase of Th2 cell development was necessary during mucosal Th2 responses, WT or TSLP−/− *naïve* mice were injected with ovalbumin (10 µg) into the airway every other day until day 5. On day 6 isolated lung draining lymph node cells were restimulated with ovalbumin and Th2 cytokines were examined in 48 hrs supernatants. While the levels of Th2 cytokines were relatively low at this early day 6 time point, IL-5 and IL-13 were significantly decreased in TSLP−/− mice compared to wild type mice (Fig. 1A). These data support the concept that TSLP is involved in early differentiation of Th2 cells. To investigate further whether the absence of TSLP in vivo impacts the airway sensitization responses, we used an adoptive transfer system with ova-specific DO11.10 TCR transgenic T cells. For the sensitization phase, naïve DO11.10 CD4 T cells were transferred into wild type or TSLP−/− mice. The mice were injected with ovalbumin (5 ug) every other day until day 5 through an intranasal route. All groups of mice that received naïve DO11.10 T cells followed by exposure to ovalbumin showed no dramatic change in airway inflammation and mucus staining in the bronchial epithelium at this early stage of differentiation (Fig. S1). However, restimulation of lymph node cells showed impaired Th2 cytokine secretion in TSLP−/− mice compared to wild type mice (Fig. 1B). These results further suggest that TSLP is required for the development of the Th2 immune response and may be a T cell associated mechanism.

To investigate the role of TSLP on already established Th2 cells, *in vitro* differentiated with IL-4 *in vitro* transgenic DO11.10 Th2 cells were transferred into either wild type or TSLP−/− mice. The mice were challenged with ovalbumin every other day until day 5 through an intranasal route. Mice that received Th2 cells followed by ovalbumin injection exhibited airway inflammation and increased mucus staining within the bronchial epithelium (Fig. 1C). Lung histology showed that mucus production and inflammation were induced at similar levels in wild type and TSLP−/− mice that received Th2 cells followed by ovalbumin injection. Similar expression levels of gob5 and muc5ac in WT and TSLP−/− mice confirmed the histology data (Fig. 1D), as these are two genes associated with goblet cell metaplasia and mucus production, respectively. We also examined the Th2 cytokines in supernatants from lung draining lymph nodes restimulated with ovalbumin. The levels of IL-4, IL-5, and IL-13 were similar between restimulated lymph node cells from wild type and TSLP−/− mice (Fig. 1E). Therefore, the absence of TSLP did not appear to impact Th2 cytokines from previously skewed T cell populations.

### TSLP Acts on DC and T Cells for Enhancing Th2 Cytokine Production

**Figure 2:**
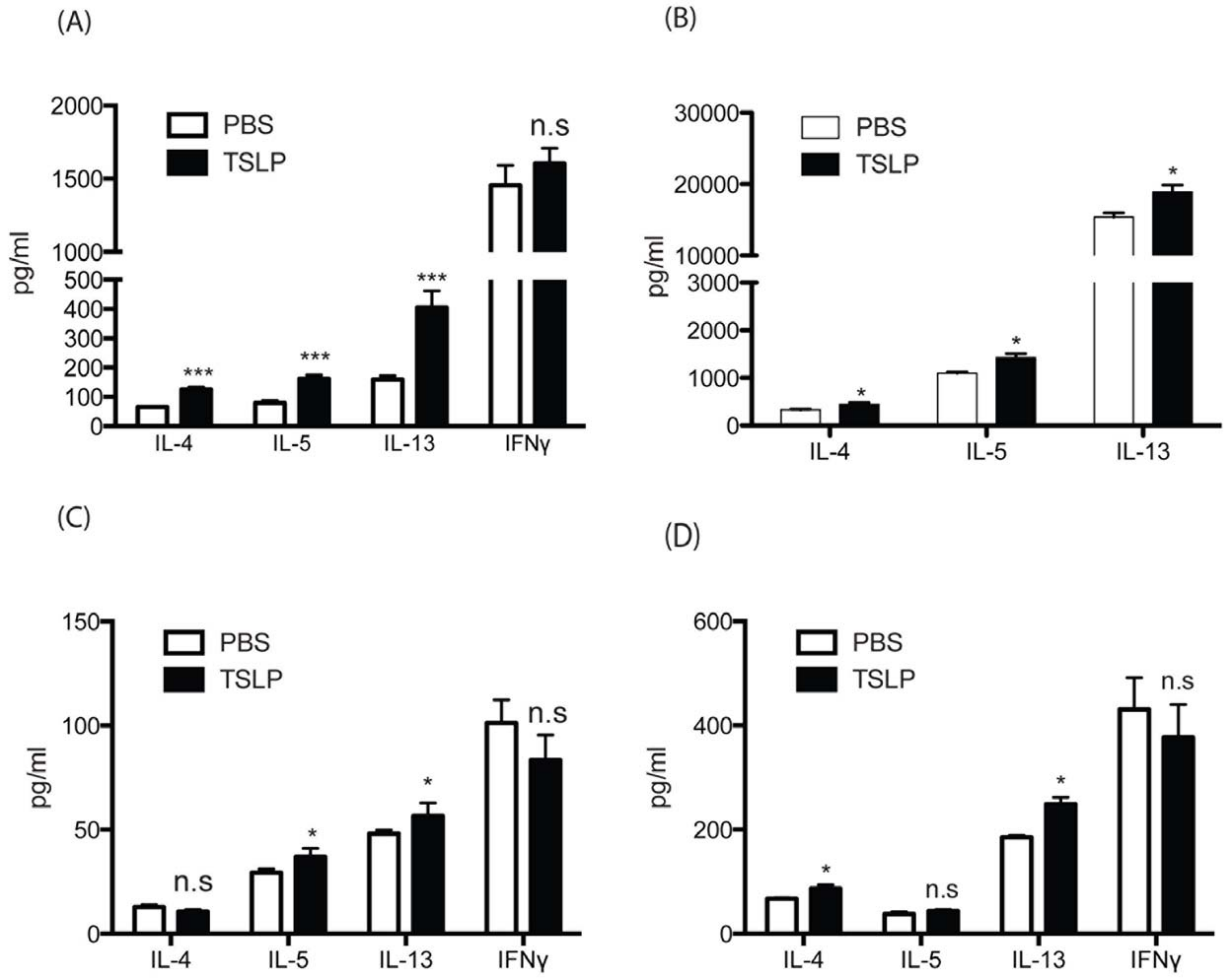
TSLP alters naïve CD4 T cell to express Th2 cytokines both on its effect on DCs and T cells. (A and B) DO11.10 CD4 T cells were cocultured with wild type *BMDCs* (A) were incubated plus ova peptides (10 ug/ml) and +/− TSLP *(15* *ng/ml)*. Supernatants were collected after 48 hrs and assayed for cytokines by Bioplex. (C) DO11.10 CD4 T cells were cultured in Th2 biased conditions for 8 days, and restimulated with ova peptides +/− TSLP for 48 hrs and supernatant were assayed for cytokines by Bioplex. (D) BMDCs or (E) lung CD11c cells were incubated with ova peptides (10 ug/ml) along with either presence or absence of TSLP (15 ng/ml) overnight. Next day DCs were washed extensively and CD4 T cells from DO11.10 spleens were added into DC cultures. After 48 hrs, supernatants were assayed for cytokines by Bioplex. Data represent mean ± SEM from 3 repeated experiments with triplicate wells.

Previous data demonstrated that TSLP induced responses are involved in the early development of Th2 immune responses [11], [12], [28], [29]. First we investigated the effect of TSLP on T cell activation during a primary stimulation when co-cultured with BMDCs. TSLP and ova peptides were added into the co-culture of DO11.10 CD4 T cells along with BMDCs in a synchronous fashion for 48 hrs. Similar to previous results [30], the TSLP treated co-culture system produced higher levels of Th2 cytokines compared to untreated cultures (Fig. 2A). Similar levels of IFNgamma, a Th1 cytokine, were observed whether or not TSLP was present. To address whether TSLP could also enhance the response of an established Th2 disease, Th2 cells were generated using DO11.10 T cells and irradiated BMDCs cultured under Th2 biased conditions for 8 days. To remove dead irradiated BMDCs on day 8, cultured cells were Ficoll-Paque gradient-separated and live T cells restimulated with freshly differentiated BMDCs incubated with ova peptides with or without TSLP. While the magnitude of change compared to primary stimulation conditions was not as impressive, the TSLP co-culture produced a significant increase of Th2 cytokines compared to co-culture without TSLP (Fig. 2B). When the expression level of TSLPR on T cells was examined by flow cytometry, Th2 skewed cells expressed much higher TSLPR on their cell surface compared to naive T cells (Fig. S2A) consistent with the previous report [30]. Thus, while the magnitude of effect of TSLP on naïve vs. Th2 skewed T cells could not be explained by the TSLPR expression level alone, it does appear that the increased expression of TSLPR on Th2 cells might present an activating response during chronic disease.

Next, to test whether TSLP alters DCs to affect the activation of CD4 T cells for cytokine expression in the murine system, we pretreated BMDCs with ova peptides together with either PBS or TSLP. After overnight incubation, DCs were extensively washed to remove OVA and TSLP and isolated ova peptide-specific naïve DO11.10 CD4 T cells were added into those BMDC cultures. After an additional 48 hours of incubation, Th2 cytokines were examined in the supernatant. IL-4 was not changed regardless of the treatment of TSLP to DCs (Fig. *2D*). However, there was a significant, but not substantial increase in IL-5 and IL-13 from the TSLP treated DC co-culture compared to untreated DC co-cultures (Fig. *2C*). TSLP has been shown to promote the ability of human DCs to polarize naïve T cells into Th2 cells by up-regulating OX40L on DCs in IL-12 deficient conditions and may contribute to their ability to drive Th2 responses. Therefore we examined the OX40L expression on DCs after TSLP treatment. Interestingly OX40L expression on DCs was only slightly increased (Fig. S3A) on TSLP treated DCs. Murine DCs express TSLPR (Fig. S2C) and respond to TSLP as CD86 was induced in TSLP-treated DCs (Fig. S3B). We also used flow cytometry sorted lung CD11c DCs from naïve mice as antigen presenting cells. The co-culture of DO.11 T cells with TSLP treated lung DCs pulsed with ovalbumin produced significantly higher levels of Th2 cytokines, IL-4 and IL-13, compared to cells from the co-culture of PBS treated lung DCs (Fig. *2D*). Thus, the enhanced Th2 cytokines generated during T cell activation in the presence of TSLP appeared to be dependent upon the presence of DCs.

### Absence of TSLP does not Impair Later Stages of Allergen-induced Responses

**Figure 3:**
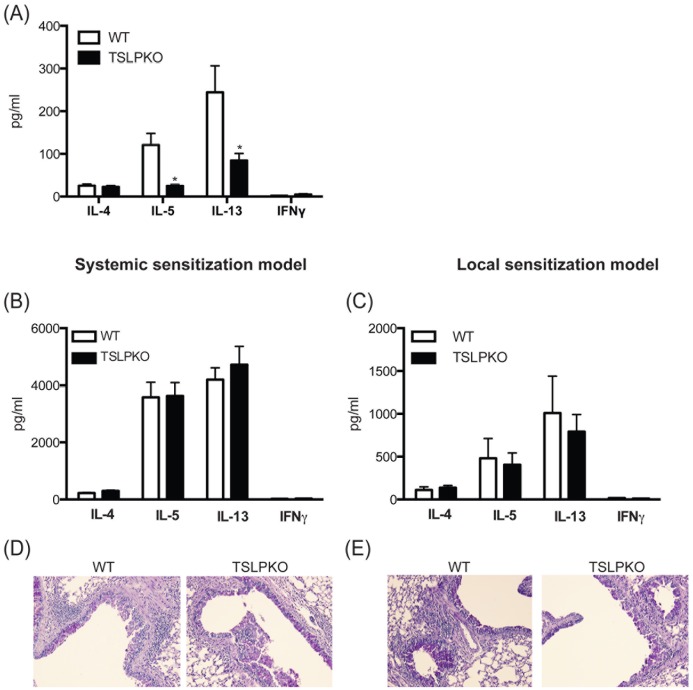
Absence of TSLP does not impair development of cockroach antigen (CRA)-induced allergic response. Mice were injected with CRA by intranasal exposure on day 1, 3, and 5. (A) On day 6, lymph node cells were restimulated with CRA and supernatants were analyzed by Bioplex. Mice were either sensitized i.p./s.c. (B and D) or i.n. (C and E) and challenged (See material and method section). Lymph nodes were taken one day after final challenge and (B and C) analyzed cytokines from allergen-restimulated lung draining lymph nodes by Bioplex. (D and E) lungs were stained with PAS. Data represent mean±SEM from 5 mice/group.

Since *in vitro* and *in vivo* data showed a critical role of TSLP primarily during Th2 cell development, we used our cockroach allergen (CRA)-induced murine model to investigate the role of TSLP using WT and TSLP−/− mice. To examine whether the presence of TSLP at the very early phase of Th2 cell development was necessary for allergen-induced mucosal Th2 responses, WT or TSLP−/− mice were injected with CRA into the airway every other day until day 5. On day 6 isolated lung draining lymph node cells were restimulated with CRA and Th2 cytokines were examined in 48 hrs supernatants. Similar to the ovalbumin model (Fig. 1A), allergen specific Th2 cytokines, IL-5 and IL-13, were decreased in TSLP−/− mice compared to wild type mice (Fig. 3A). These data further support the role of TSLP at early stages of differentiation of Th2 cells in allergen-induced airway disease. To further investigate the role of TSLP on the allergic response *in vivo*, we used two different shorter-term allergic models compared to a standard *chronic allergic* models and sensitized animals in either a systemic or local pulmonary route. For the first model, we sensitized mice systemically with a combination of intraperitoneal and subcutaneous injections of allergen emulsified in IFA and challenged with allergens on day 8 and day 10 through an intranasal route. For the second model, mice were injected with allergen every other day via an intranasal route from day 0 to day 10. Similar to the DO11.10 model above with previously skewed Th2 cells, the levels of allergen-specific Th2 cytokines from restimulated lung draining lymph nodes in each of these models was not significantly different between TSLP−/− and wild type mice (Fig. 3B and 3C). Histologic examination showed a similar appearance of mucus production in the lungs of wild type and TSLP−/− mice (Fig. 3D and 3E). Interestingly, lung CD4 T cells in allergic mice demonstrated that activated CD69^+^CD4 T cells expressed more TSLPR than CD69^-^CD4 T cells (Fig. S2B). Thus, TSLP plays an important role at the initial stage of Th2 differentiation, but there appears to be no effect at the later stage of allergic response when Th2 differentiation has already been established.

**Figure 4:**
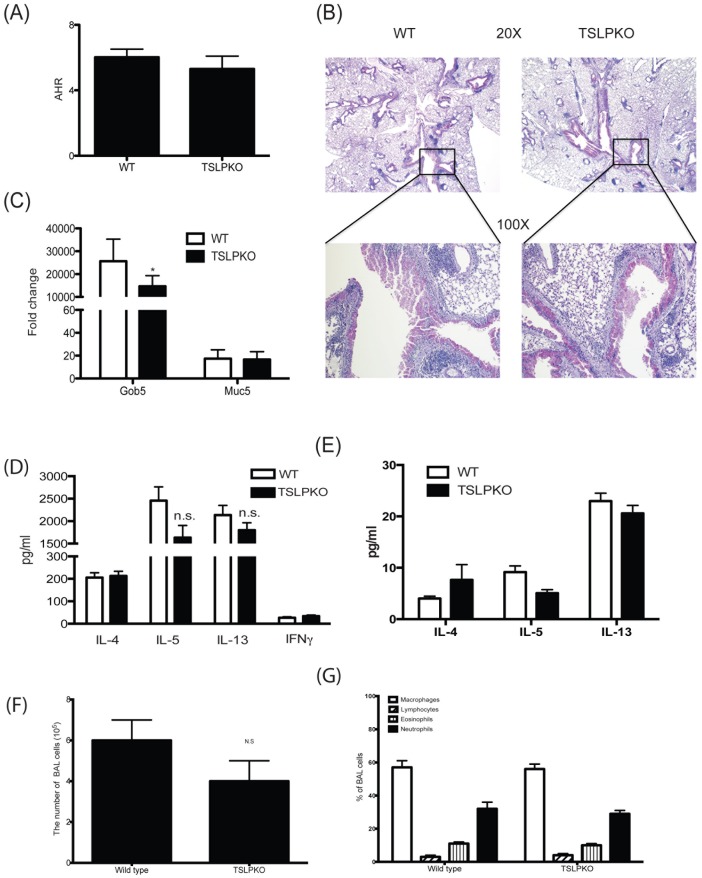
Absence of TSLP does not impair cockroach allergen induced chronic allergic response. After repeated challenges of allergen (see Materials and Methods) over a 4 week period animals were examined for their allergic responses. (A) Airway responses were measured after one dose of methacholine. Data are represented as the peak airway resistance in H2O/ml/s±SEM. (B) Lungs were taken one day after final challenge and were stained with PAS and (C) assayed for gob5 and muc5ac expression by real-time PCR. Fold changes were calculated upon comparison to control lungs from naïve animals. (D) Analysis of cytokines from allergen-restimulated lung draining lymph nodes was assessed by Bioplex. (E) Supernatants from lung homogenates were assayed for cytokines by Bioplex. (F and G) BAL fluid was collected 24 hours after the last CRA challenge and was analyzed (F) for the total number of cells recovered by BAL and (G) for the percentage of each cell type. Data represent mean±SEM from 5 mice/group.

In order to determine if there might be a role for TSLP in more severe allergen-induced disease, mice were sensitized with a more chronic exposure with 6 instillations of allergen into the airways to initiate a persistent response over several weeks. Initial assessment of lung physiology using methacholine challenge and measurement of airway hyperreactivity in ventilated mice demonstrated that there was no difference in AHR between wild type and TSLP−/− mice (Fig. 4A). Moreover, while lung histology appeared to show slightly more mucus in wildtype compared to TSLP−/− mice, both wild type and TSLP−/− mice showed significant cellular recruitment into the lungs and the presence of excessive mucus production and goblet cells (Fig. 4B). Quantitative PCR of the mucus-associated genes, gob5 and muc5ac, in the lungs of mice reflected the histology data demonstrating very significant increases in the expression pattern in both the WT and TSLP−/− mice compared to untreated mice. However there was a decrease in the level of gob5 expression in TSLP−/− mice potentially demonstrating an alteration in mucus expression (Fig. 4C). However, the levels of allergen-specific IL-4, IL-5, and IL-13 from lymph nodes were not significantly different in TSLP−/− mice compared to wild type mice, although IL-5 and IL-13 were reduced (Fig. 4D). Similar to lymph node data, there was no significant difference in Th2 cytokines in lung homogenates from wild type and TSLP−/− mice (Fig. 4E). Analysis of BALF showed a modest decrease in cell number in TSLP−/− mice, but did not reach significance (Fig. 4F) and there was no difference in the ratio of different cell types in BALF from wild type and TSLP−/− mice (Fig. 4G). Thus, similar to the less severe, shorter-term models of allergen/antigen exposure, deficiency of TSLP appeared to have little impact on the immune responses in this model of chronic allergen exposure.

### Blockade of TSLP does not Change Allergic Airway Response or Th2 Immunity

**Figure 5:**
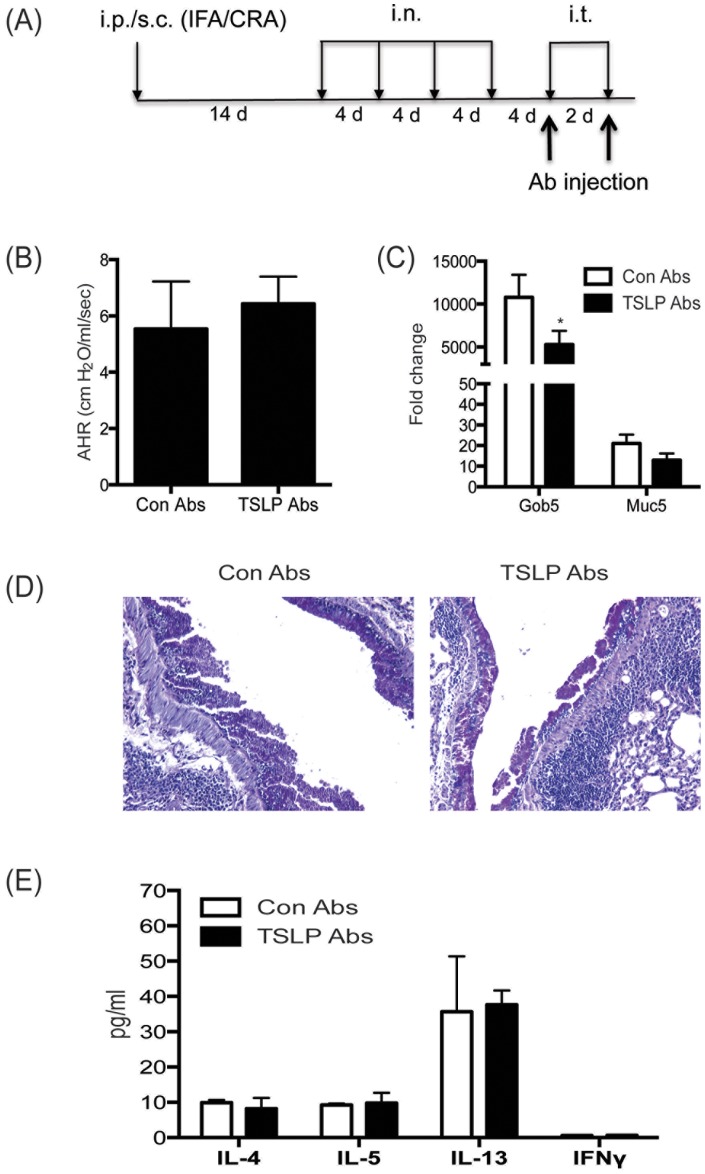
In vivo blockade of TSLP signaling at the late phase of allergic diseases does not affect cockroach allergen-induced allergic reaction. (A) Model outline and timing of Ab injections during chronic model of cockroach antigen-induced allergic lung disease was based upon applying anti-TSLP (M702) in a therapeutic format at the end of the challenge period. (B) Airway responses were measured after one dose of methacholine in ventilated and anesthetized animals. Data are represented as the peak airway resistance in H_2_O/ml/s±SEM. Lungs were taken one day after final challenge and were (C) assayed for Gob5 and Muc5ac expression and (D) stained with PAS. (E) Analysis of cytokines from allergen restimulated lung draining lymph nodes by Bioplex. Data represent mean±SEM from 5 mice/group. Fold changes were calculated upon comparison to control lungs from naïve animals.

Since TSLP−/− mice may have been able to compensate for the absence of TSLP in an ongoing response the above results may not have fully evaluated whether TSLP would be a good target during later stage allergic airway disease. To further examine the role of TSLP on an established Th2 environment and to determine if TSLP could be a target for therapy, our chronic allergen model was utilized along with treatment with monoclonal anti-TSLP antibody. Allergic mice were injected with anti-TSLP abs, as previously described [31], just prior to each of the two final allergen challenges to neutralize TSLP within the allergen-induced model (Fig. 5A). Consistent with the findings in the TSLP−/− mice, TSLP ab treated mice did not display a difference in AHR (Fig. 5B). However, the anti-TSLP treated animals did demonstrate a modest, but significant decrease in gob5 gene expression in the lungs compared to control ab treated mice (Figure 5B). When lung pathology was examined in histologic sections there was not a clear difference in the appearance of the disease intensity (Fig. 5C). However, when lung draining lymph node cells were re-stimulated with allergen and supernatants were analyzed for Th2 cytokines the levels of IL-4, IL-5, and IL-13 were similar in both control and anti-TSLP ab treated mice (Fig. 5D). Thus, TSLP neutralization appeared to have a modest effect on disease pathology but did not appear to be necessary for an already established allergic, Th2 cytokine mediated response.

**Figure 6:**
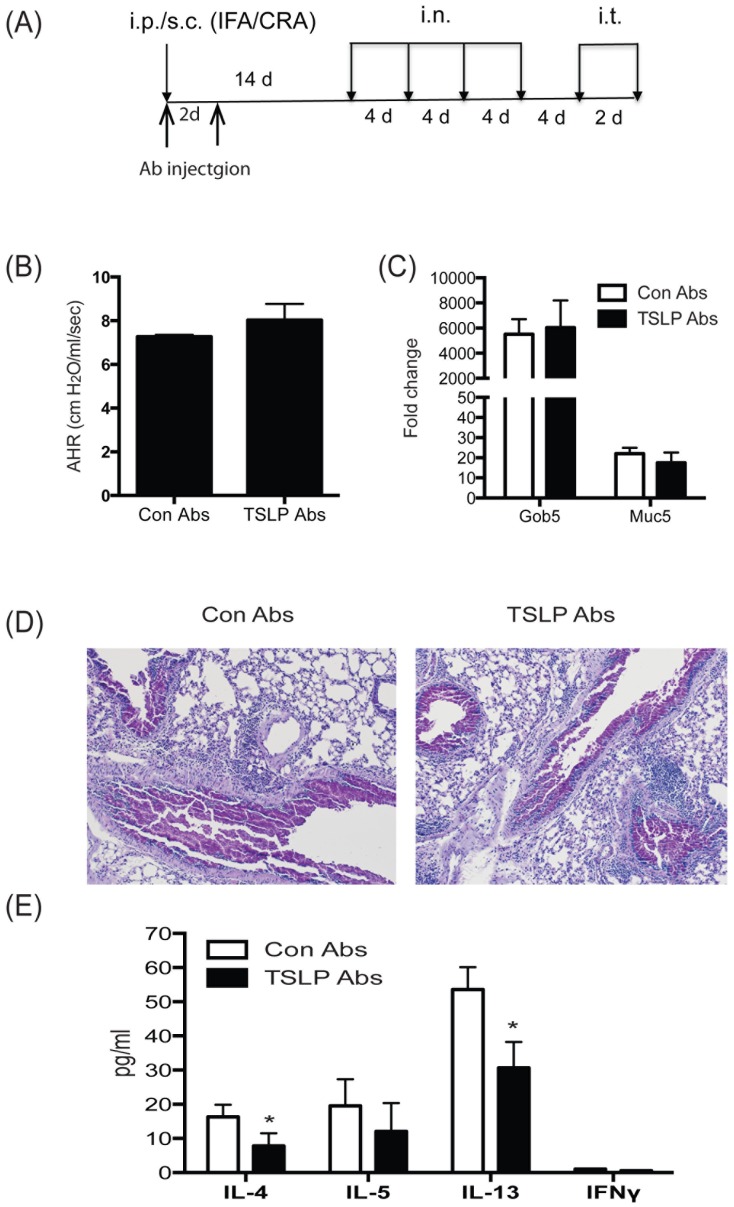
In vivo blockade of TSLP signaling at the induction phase of allergic diseases does not attenuate cockroach allergen-induced airway reaction. (A) Model outline and timing of Ab injections during the chronic model of cockroach antigen-induced allergic lung disease. (B) Airway responses were measured after one dose of methacholine in ventilated and anesthetized animals. Data are represented as the peak airway resistance in H_2_O/ml/s±SEM. Lungs were taken one day after final challenge and were (C) assayed for Gob5 and Muc5ac mRNA expression by QPCR and (D) stained with PAS. (E) Analysis of cytokines from 48 hr supernatants of allergen re-stimulated lung draining lymph nodes by Bioplex. Data represent mean±SEM from 5 mice/group. Fold changes in QPCR analyses were calculated upon comparison to control lungs from naïve animals.

Next, anti-TSLP antibodies were injected on day 0 and 3 of sensitization to examine the role of TSLP at the early phase of our chronic allergen model (Fig. 6A). Consistent with the result in our previous results with TSLP−/− mice (Fig. 3 and 4), TSLP ab treated mice did not display a difference in AHR (Fig. 6B) or the expression of mucus related genes (Fig. 6C). Histologic examination showed a similar appearance of mucus production in the lungs of control and anti-TSLP ab treated mice (Fig. 6D). However, when lung draining lymph node cells were re-stimulated with allergen, the levels of IL-4 and IL-13 did demonstrate a modest, but significant decrease in anti-TSLP ab treated mice compared to control ab treated mice (Fig. 5D). Thus, similar to results observed in both the shorter-term and chronic allergen models with TSLP −/− mice (Fig. 3 and 4), TSLP neutralization appeared to have little impact on the ultimate outcome of the pathologic responses in this model of chronic allergen exposure.

## Discussion

Previous work has suggested that TSLP and TSLPR signaling is critical for the generation of the Th2 response [6], [32]. Studies using human monocyte derived cells demonstrated that TSLP-treated DCs induce the differentiation of naïve T cells into a Th2 phenotype, partly through induction of OX40L expression on DCs [33] [13], while the overexpression or ablation of TSLP in specific organs or specific types of cells demonstrated that TSLP is indispensible for generation of Th2 immunity in allergic responses [5] [34]–[35]. Other studies have shown that mouse DCs increased CCL17, a Th2 associated chemokine, in response to TSLP [5]. Because of its role in Th2 immunity, TSLP has become a strong candidate as a therapeutic target in asthmatic patients. In this study, it was demonstrated that TSLP enhanced Th2 skewing was operative through its effects on DCs and T cells. This was examined using TSLP-stimulated BMDC from WT mice and isolated WT pulmonary DC populations as antigen presenting cells, verifying previous data regarding the importance of DC. The apparent direct effects on increased Th2 cytokine was not due to higher proliferation of T cells in the TSLP treated co-culture since there was no increase of cell number during the 48 hr co-culture period (data not shown). Likewise, Lu et al. showed that TSLP failed to promote the proliferation of human CD4 T cells with CD3/CD28 stimulation [36]. Interestingly, the latter study demonstrated that TSLP could promote the proliferation of human CD4 T cells when they were stimulated with CD3/CD28 in the presence of myeloid DCs suggesting that in humans DCs also have a prominent role. When our studies utilized adoptive transfer experiments with naïve DO11.10 CD4 T cells, TSLP−/− mice showed impaired development of Th2 cells compared to wild type mice. These results were consistent with previous studies using adoptive transfer of TSLPR sufficient T cells in TSLPR−/− mice, which allowed the restoration of airway inflammation and food allergy [10] [37] together suggesting that TSLP also has a direct role on T cells. Recently a study using anti-TSLPR-immunoglobulin treatment supported a role for TSLP during Th2 sensitization [38]. The relative importance of TSLP on each cell type observed in the present study may vary in different Th2 associated models, with specific DC subsets, and/or different species (rodent vs. human). In addition, the site of allergic response may also be differentially affected by TSLP as allergic skin and gastrointestinal responses can be significantly altered by the presence or absence of TSLP [37], [39], [40], [41]. The TSLP−/− mice used in the present study were recently utilized in a skin sensitization model for examining pulmonary responses and the findings in this latter study also suggested that TSLP was dispensable for airway challenge at later time points after skin sensitization [23]. One aspect that the present study did not pursue in detail was the role of TSLP on generation of Treg cells [20], [22], [42]. While we have not observed a distinct difference in Treg numbers in our studies (Fig. S4A), it does not rule out an altered function in the knockout animals. Thus, there may be several mechanisms that are operative in the absence of TSLP that could contribute to the overall response.

Another important finding of this study came from the investigation of the role of TSLP during the initiation of Th2 responses vs. established allergic disease. Together, those data indicate that TSLP did not appear to have a prominent effect during established Th2-mediated disease. A recent study showed that TSLP enhanced the production of Th2 cytokines upon re-stimulation of Th2 skewed T cells [30], which was largely verified in the present study. Data from adoptive transfer of ova-specific T cells and ab-blocking experiments in the present study demonstrated that TSLP is dispensable within the established Th2 environment. However, there was an alteration in gob5 expression in the longer-term allergen challenge model in both the TSLP−/− and anti-TSLP treatment studies identifying a potential benefit to targeting TSLP in established disease settings for development of mucus overexpression. In previous studies, a mouse model of schistosomiasis, which has Th2 cytokine driven pathology, showed that TSLP signaling was involved in the development of polarized Th2 cytokine response during a primary response, but TSLP was dispensable for the formation of Th2-dependent pathology during a chronic *S. mansoni* infection [43]. Thus, there appear to be compensatory mechanisms available to the immune system during chronic antigen exposure that allows the Th2 response to develop. This may be an especially important adaptation during infectious disease conditions where the Th2 response is required for effective protective responses, as in the case of chronic helminth infections [44]. One aspect that we did not test in the present study is whether TSLP could be a target in established Th2 disease upon a viral exacerbation. Recent studies have identified that RSV, in particular, is a strong inducer of TSLP that may help to initiate a local pulmonary pathologic response [45], [46], [47], [48].

Multiple mechanisms are involved in maintenance of Th2 immunity. The emergence of an important triad of cytokines, TSLP, IL-25 and IL-33, that play a role in the initiation and maintenance of a Th2 response has focused many on the interaction of these cytokines [49], [50], [51], [52]. It was found that treatment of a lung epithelial cell line with IL-25 increased TSLP expression. Furthermore, two important Th2 innate cells, activated eosinophils [53] and basophils [54], secrete bioactive IL-25, which could up-regulate TSLP, indicating cross-talk between immune cells and structural cells related to allergic disease. Interestingly, IL-33 treatment also increased the expression of TSLP and TSLPR in the colons of *Trichuris*-infected mice [55]. Relatedly, in the present study allergic mice appeared to have increased IL-25 and IL-33 expression in the lung during allergen exposure (Fig. S4). However, there was no difference of expression of these genes between wild type and TSLP −/− mice perhaps suggesting that these cytokines may provide a compensatory mechanism during established disease. Previous studies indicated that basophils can produce TSLP at the initiation of type-2 immune responses in a papain-immunized mouse allergic model [56]. Several studies have suggested that basophils are transiently recruited to draining lymph nodes during allergic disease [15], [57], [58]. Thus, basophils may play an important role in early Th2 differentiation events in tissue and lymph nodes since they are a source of TSLP, as well as IL-4, that would directly impact T cell differentiation. In addition, TSLP appears to directly influence basophil development and activation. Recently, Siracusa et al. demonstrated that TSLP influenced allergic susceptibility by regulating basophil hematopoiesis [15]. Mice overexpressing TSLP exhibited an increase in the number of basophils that was directly related with systemic production of Th2 cytokines and the development of Th2 cytokine-associated intestinal inflammation. Furthermore, adoptive transfer of TSLP-elicited basophils into *Trichuris muris* infected TSLPR−/− mice partially restored protective Th2 cytokine responses. Interestingly, in the latter study TSLP-elicited basophils produced IL-4 after activation with IL-33, suggesting cooperation of TSLP and IL-33 for Th2 response development. A stronger link of TSLP and disease can be found in patients with atopic dermatitis with an increase of TSLP level [59] and increased frequency of human CD4^+^Th2 memory cells expressing the prostaglandin D2 receptor (CRTH2) [59], [60], suggesting TSLP may be involved in maintenance of Th2 memory pools. Further, Wang et al. reported that IL-25 receptor was highly expressed on CD4^+^ Th2 memory cells and the IL-25 receptor expression on this population was up-regulated after culturing with TSLP-activated DCs. Indeed, IL-25 co-stimulates the expansion of Th2 memory cells and enhances their Th2 cytokine production along with increased expression of Th2 transcription factors [54], [59]. Other studies also demonstrated that OX40L and IL-25, which is directly upregulated on TSLP-stimulated DCs, plays an important role in activation of allergic responses [59] [52] [61]. These recent findings suggest that there is a complex interplay between these innate molecules and cells that produce Th2 cytokines that may subsequently mediate disease associated inflammation, such as in asthma, atopy, and helminth infections. Understanding the biologic interaction between these innate molecules and their cellular sources related to the maintenance of allergic responses could provide novel therapeutic approaches for Th2 mediated diseases. However, our data in murine models suggest that TSLP is primarily involved in the generation of Th2 responses and short-term therapeutically targeting of TSLP using mono-therapy protocols may have limited value in a purely Th2 cytokine mediated disease.

## Supporting Information

Figure S1 **Histologic examination of mucus expression by Periodic acid Schiff staining.** Lungs from mice received DO11.10 CD4 T cells plus ovalbumin were taken one day after final challenge and were stained with PAS to allow examination of goblet cell hyperplasia differences. Histology is representative of 5 mice/group.
(TIF)

Figure S2 **Flow cytometry analysis of TSLPR expression on T cells and dendritic cells.** (A) Naïve T cells (dashed line) and Th2 cells (solid line) were stained with anti-TSLPR abs. (B) lung CD69^+^CD4^+^T cells (solid line) and CD69^-^CD4^+^T cells (dashed line) from CRA-induced allergic mice were stained with TSLPR abs. (C) BMDCs and lung CD11c cells were stained with anti-TSLPR abs (R&D systems, Minneapolis, MN) (solid line) or with isotype control abs (dashed line). Alveolar macrophages were excluded using their auto-fluorescent properties when lung CD11c cells were sorted. Data represents analysis of 5 mice/group.
(TIF)

Figure S3 **Flow cytometry analysis of TSLP inducible costimulatory molecules on DCs.** BMDCs were stimulated with TSLP (15 ng/ml) for 24 hours and stained with (A) OX-40L (Biolegend, San Diego, CA) and (B) CD86 (BDbiosciences, San Diego, CA). PBS treated BMDCs (dashed line) and TSLP treated BMDCs (solid line). Data is representative of 3 repeat experiments.
(TIF)

Figure S4 **Expression of Treg cells and innate cytokines IL-25 and IL-33 were not altered by TSLP.** (A) Lymph node cells from chronically allergic wild type and TSLP−/− mice were analyzed for regulatory T cells with staining of markers, CD4, CD25, and FoxP3 (Biolegend) by flow cytometry. (B) Lungs from chronically allergic mice were assayed for IL-25 and IL-33 expression by real-time PCR demonstrating increased expression when compared to lung mRNA from non-allergic mice. Data represents mean ± SE from 5 mice/group.
(TIF)
