# Supplementary figures and images for: TSLP Promotes Induction of Th2 Differentiation but Is Not Necessary during Established Allergen-Induced Pulmonary Disease
Source: PLoS One. 2013 Feb 20;8(2):e56433. doi: 10.1371/journal.pone.0056433 (PMC3577905; doi:10.1371/journal.pone.0056433)

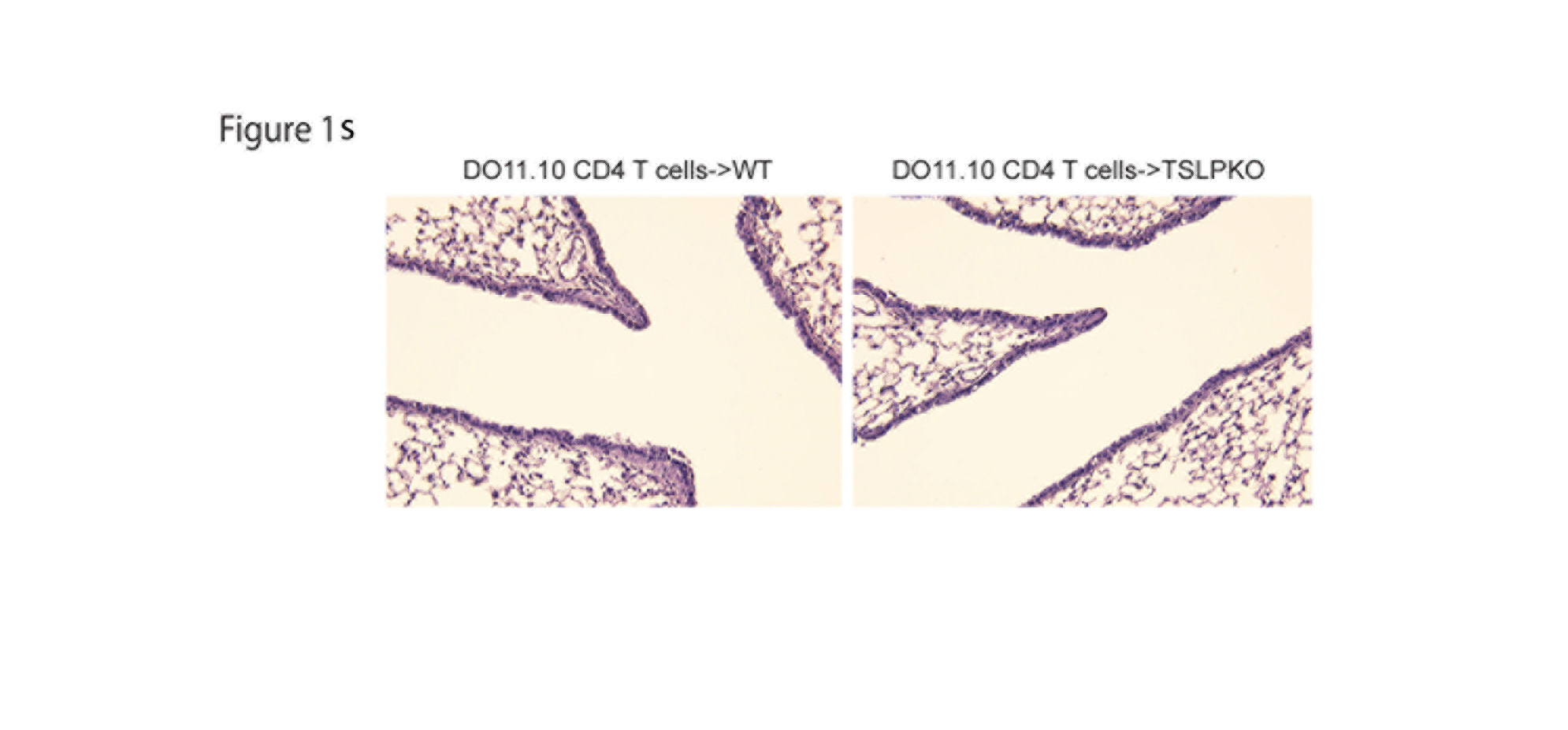

Supplement: Figure S1 — Histologic examination of mucus expression by Periodic acid Schiff staining. Lungs from mice received DO11.10 CD4 T cells plus ovalbumin were taken one day after final challenge and were stained with PAS to allow examination of goblet cell hyperplasia differences. Histology is representative of 5 mice/group. (TIF) [file pone.0056433.s001.tif]

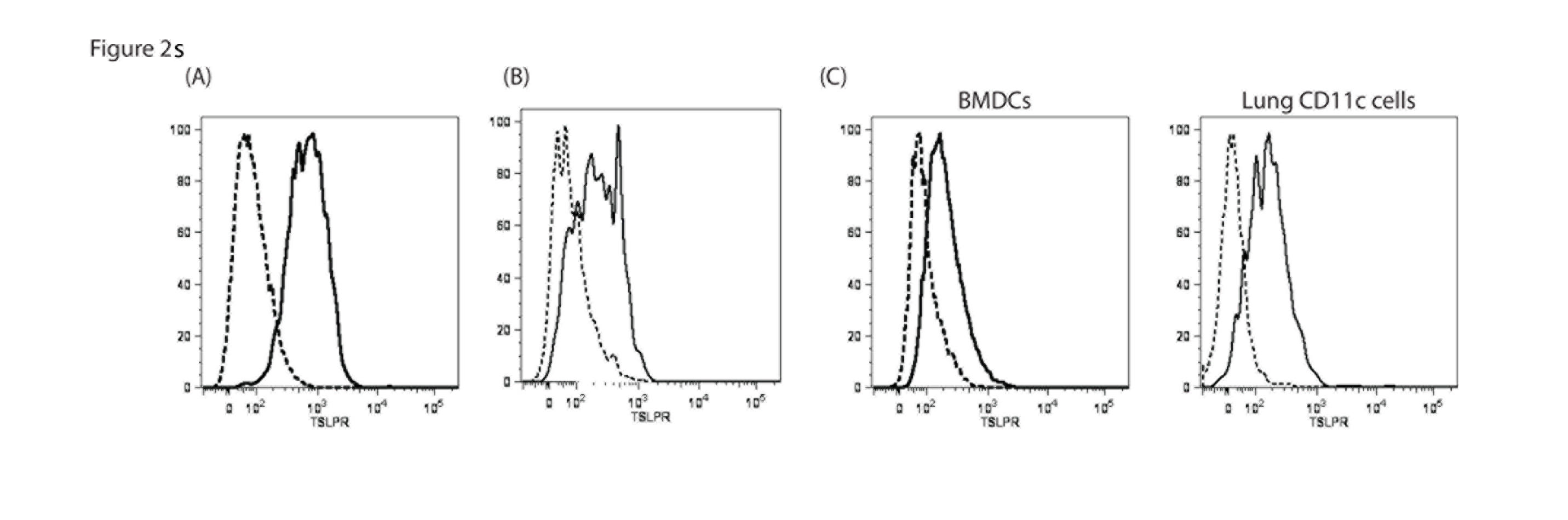

Supplement: Figure S2 — Flow cytometry analysis of TSLPR expression on T cells and dendritic cells. (A) Naïve T cells (dashed line) and Th2 cells (solid line) were stained with anti-TSLPR abs. (B) lung CD69+CD4+T cells (solid line) and CD69-CD4+T cells (dashed line) from CRA-induced allergic mice were stained with TSLPR abs. (C) BMDCs and lung CD11c cells were stained with anti-TSLPR abs (R&D systems, Minneapolis, MN) (solid line) or with isotype control abs (dashed line). Alveolar macrophages were excluded using their auto-fluorescent properties when lung CD11c cells were sorted. Data represents analysis of 5 mice/group. (TIF) [file pone.0056433.s002.tif]

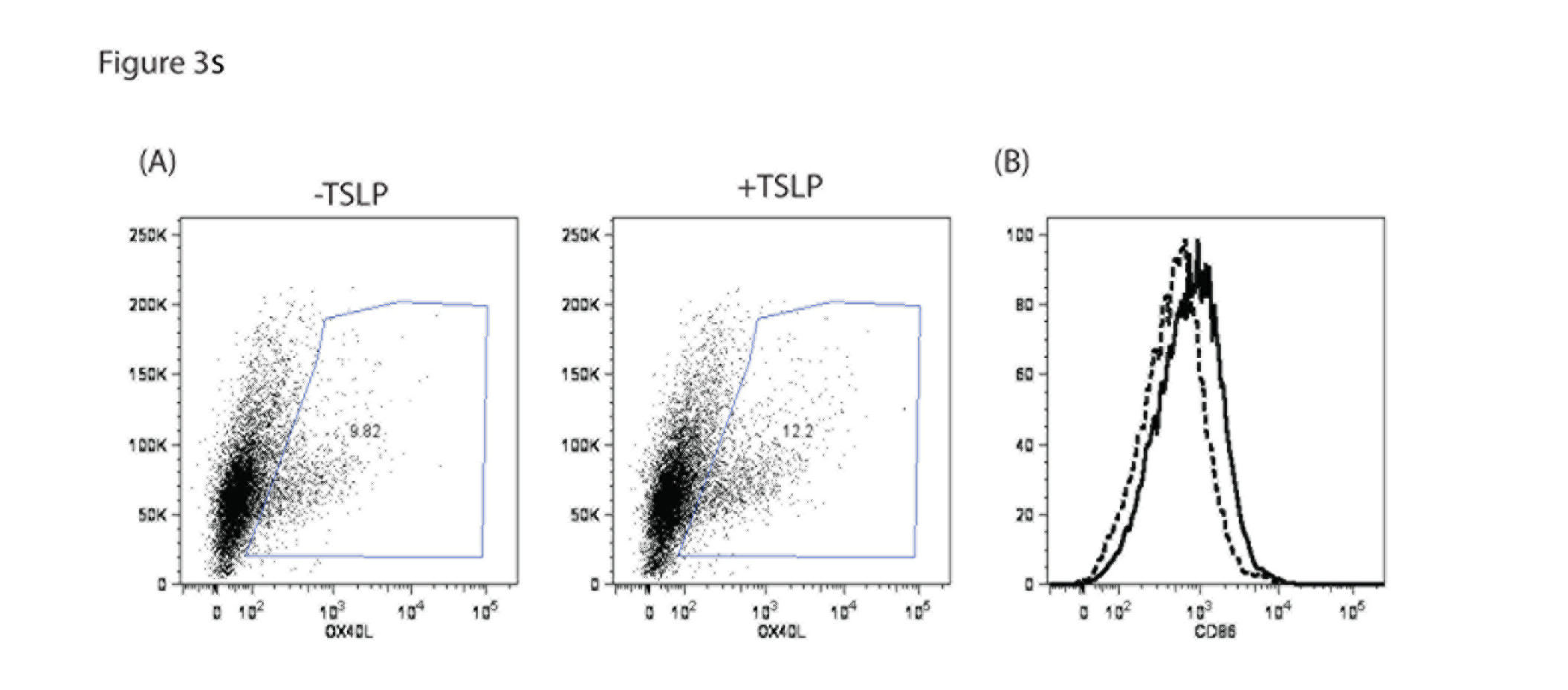

Supplement: Figure S3 — Flow cytometry analysis of TSLP inducible costimulatory molecules on DCs. BMDCs were stimulated with TSLP (15 ng/ml) for 24 hours and stained with (A) OX-40L (Biolegend, San Diego, CA) and (B) CD86 (BDbiosciences, San Diego, CA). PBS treated BMDCs (dashed line) and TSLP treated BMDCs (solid line). Data is representative of 3 repeat experiments. (TIF) [file pone.0056433.s003.tif]

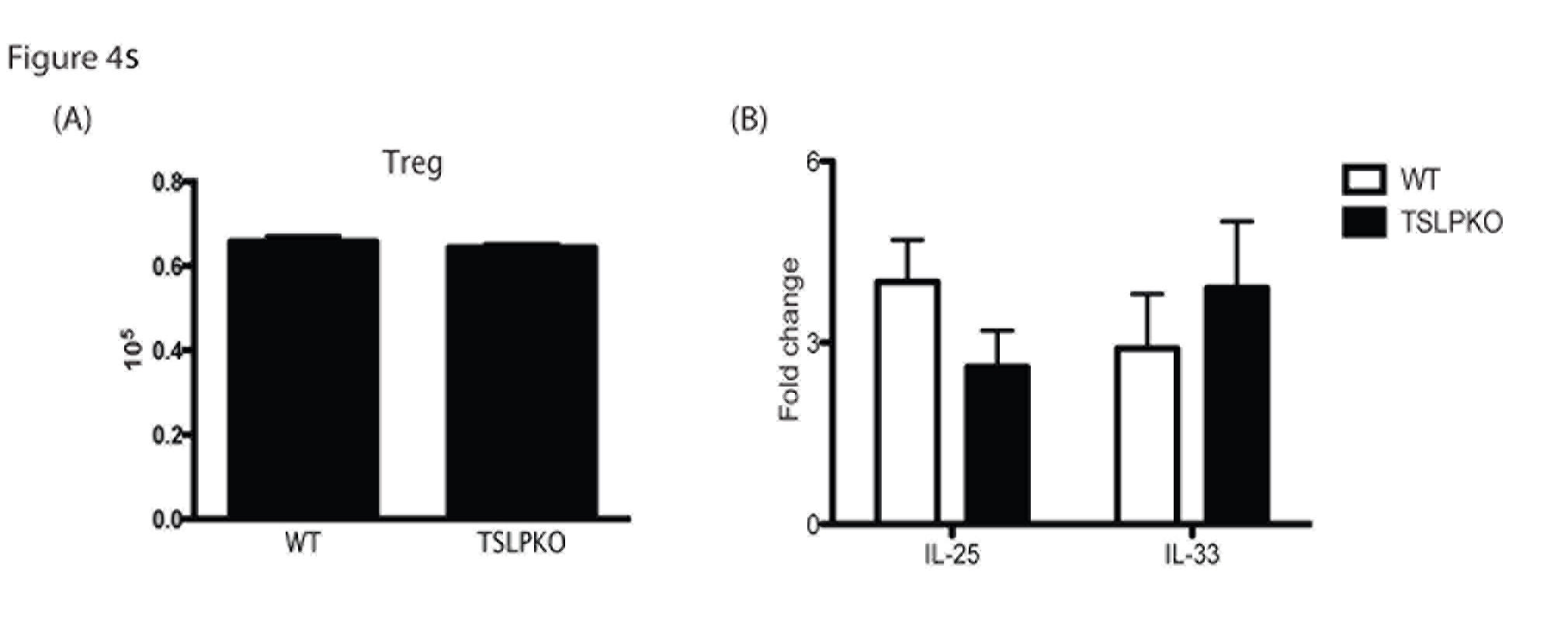

Supplement: Figure S4 — Expression of Treg cells and innate cytokines IL-25 and IL-33 were not altered by TSLP. (A) Lymph node cells from chronically allergic wild type and TSLP−/− mice were analyzed for regulatory T cells with staining of markers, CD4, CD25, and FoxP3 (Biolegend) by flow cytometry. (B) Lungs from chronically allergic mice were assayed for IL-25 and IL-33 expression by real-time PCR demonstrating increased expression when compared to lung mRNA from non-allergic mice. Data represents mean ± SE from 5 mice/group. (TIF) [file pone.0056433.s004.tif]
